# Robust Self-Adaptation Fall-Detection System Based on Camera Height

**DOI:** 10.3390/s19173768

**Published:** 2019-08-30

**Authors:** Xiangbo Kong, Lehan Chen, Zhichen Wang, Yuxi Chen, Lin Meng, Hiroyuki Tomiyama

**Affiliations:** 1 Graduate School of Science and Engineering, Ritsumeikan University, Kyoto 525-8577, Japan; 2 Department of Electronic and Computer Engineering, College of Science and Engineering, Ritsumeikan University, Kyoto 525-8577, Japan

**Keywords:** fall detection, self-adaptation, camera height, practical

## Abstract

Vision-based fall-detection methods have been previously studied but many have limitations in terms of practicality. Due to differences in rooms, users do not set the camera or sensors at the same height. However, few studies have taken this into consideration. Moreover, some fall-detection methods are lacking in terms of practicality because only standing, sitting and falling are taken into account. Hence, this study constructs a data set consisting of various daily activities and fall events and studies the effect of camera/sensor height on fall-detection accuracy. Each activity in the data set is carried out by eight participants in eight directions and taken with the depth camera at five different heights. Many related studies heavily depended on human segmentation by using Kinect SDK but this is not reliable enough. To address this issue, this study proposes Enhanced Tracking and Denoising Alex-Net (ETDA-Net) to improve tracking and denoising performance and classify fall and non-fall events. Experimental results indicate that fall-detection accuracy is affected by camera height, against which ETDA-Net is robust, outperforming traditional deep learning based fall-detection methods.

## 1. Introduction

Elderly individuals (over 65 years old) represent the fastest growing segment of the population worldwide [1]. In China, the percentage of elderly individuals was 8.87% in 2010 and is expected to quadruple between 2015 to 2050 [2]. In the United States, this was 13% in 2010 and is expected to reach 20.2% by 2050. In Europe, this was 22.5% in 2005 and is expected to reach 30% by 2050 [2]. In Japan, this was already 27.7% in 2017 and will reach 35.3% by 2040 [3]. Worldwide, the population of elderly individuals over 80 years old was 126.5 million in 2015 and is expected to be more than triple that by 2050, increasing to 446.6 million [1]. According to the World Health Organization, 28%–35% of elderly individuals have an accident involving a fall each year [4]. According to the National Safety Council, 21,600 Americans lost their lives from falling in 2007 and over 80% were elderly [5]. Globally, accidents involving falls, which are the second leading cause of unintentional death, are considered one of the most hazardous incidents for the elderly, with over 424,000 deaths occurring in 2008 [4]. Although many falls do not cause physical injuries, 47% of the elderly who have fallen cannot stand up without assistance [6]. If a solitary elderly individual falls, he or she may be lying on the floor for a long time without any help. Therefore, a fall-detection method that can automatically detect a fall in real time and send alerts to certain caregivers (such as family members, hospitals or health centers [7]) is important for solitary elderly individuals as well as playing an important part in the health care system for the elderly [8].

Fall-detection methods are roughly categorized into four groups, that is, radio-wave based, wearable/mobile-device based, pressure-sensor based and vision based. Vision-based methods are considered promising [9]. Although such methods have been studied, many have limitations in terms of practicality [10]. Some methods [11,12,13,14] establish a background model and use an image-subtraction method to segment the individual then extract features of that individual to detect a fall. However, such image-difference-based methods fail if the layout or lighting of the room changes, for example, switching lights on or changing the lighting level. The study in Reference [15] also used a dual subtraction method (background subtraction and frame subtraction) to determine the effect of moved furniture on fall-detection accuracy but the effect of light has remained a problem. Three-dimensional (3D) molding [9,16] and deep-learning [17,18] methods provide a good solution to this problem since they are robust against light conditions. However, they cannot be used in dark rooms. Few studies used infrared cameras instead of RGB cameras [19].

A depth camera/sensor [20,21] is independent of illumination and many fall-detection methods have benefited from depth-based algorithms [22]. Previous studies [23,24,25,26] used the 3D coordinates of the skeleton as features to successfully detect falls. However, these methods heavily depend on the skeleton-detection results from Kinect SDK or OpenNI, which are not reliable [8,22]. Previous studies [27,28,29] analyzed the 2D or 3D shape of humans to detect falls. However, there are problems with such shape-based methods. If an individual falls on the floor, the depth information of the floor and that of the individual are very similar, according to previous studies [8,22] and our experimental results. This results in many tracking loss or wide-ranging noise. This study proposes Enhanced Tracking and Denoising Alex-Net (ETDA-Net) to solve this problem. The experimental results indicate that ETDA-Net outperforms traditional deep-learning-based methods (LeNet, AlexNet and GoogLeNet).

Due to differences in rooms, users may set a camera or sensors at the same height. Does camera height affect fall-detection accuracy? Few studies take this into consideration [26,30]. Moreover, few open data sets for fall detection are available to analyze the effect of camera height. The data set created in Reference [12] represents various falls in different directions. That created in Reference [13] recorded numbers of daily activities and falls in different directions. The data set created Reference [31] is a well-constructed data set and images taken in different rooms, for example offices and coffee rooms, are provided. However, only a single camera was used for this data set and the camera height is not mentioned for their experiments. The data set created in Reference [32] includes depth images but there are only 21,499 images. That created in Reference [33] is a well-constructed data set and 12 people participated in their experiment. However, the camera was set at only one height, which was too low to avoid occlusion problems. The data set created in References [14,34] is also well-constructed and includes 30 fall videos and 40 videos of daily activities. Synchronization and accelerometer data were also recorded. However, the camera was set at a very low position, which may be occluded by furniture (although another camera was placed on the ceiling, it only recorded fall videos). Thus, this study creates a data set and analyzed how the camera affects fall-detection accuracy. In this data set, 16 types of daily activities (walking with a stick, sitting on a chair, sitting on the floor, using a smartphone, drinking water, etc.) and 16 types of falls (facing left, right, floor, ceiling, with/without leg bending, etc.) are carried out in eight directions and taken with the depth camera at five different heights. Eight people participated in the experiment and about 350,000 images (frames) are included in our data set. Not only depth images but also human segmentation results are provided, which will be convenient for comparative analysis.

From the experimental results, the proposed ETDA-Net provides better tacking and denoising performance than Kinect SDK and is robust against camera height. It automatically detects camera height and selects the most suitable model (trained using the images taken at the same height ) to detect a fall. The results also indicate that even if the camera height is not calculated correctly, ETDA-Net still provides a good detection rate.

The contributions of our study are as follows:

- This study analyzes how camera height affects fall-detection accuracy, which has rarely been studied before.
- This study proposes a self-adaptation fall-detection method that automatically calculates camera height and selects the most suitable detection model.
- Depth-image-based person segmentation by using Kinect SDK has been widely used in previous studies but the tracking and noise problem were rarely addressed. The proposed ETDA-Net solves this problem.
- This study creates a data set consisting of 350,000 images, more than those used in related studies. This data set includes various daily actives and falls. Each action is done in eight different directions and recorded using a depth camera at five different heights. The human-segmentation results are also included in this data set, not only for this research but also for comparative analysis.

The rest of this paper is organized as follows—Section 2 reviews related works. Section 3 introduces our fall-detection data set. Section 4 presents the proposed fall detection system and the experimental results are shown in Section 5. Finally, Section 6 concludes this paper.

## 2. Related Work

Fall-detection methods are roughly categorized into four groups—radio-wave based, wearable/mobile-device based, pressure-sensor based and vision based.

### 2.1. Radio-Wave-Based Methods

Radio-wave-based methods are roughly categorized into two types: traditional and wireless. The major advantages of these methods are non-intrusive sensing and privacy preservation.

Radar-based fall-detection methods have a strong penetrating ability and are robust against environmental factors such as illumination and humidity. Reference [35] proposed a dual-band radar-based fall-detection method. By optimizing the full ground plane and small element spacing, high mutual coupling is reduced by at least 10 dB. The effective distance with this method is 4 m at 0 degrees. However, only limited postures can be detected with the radar and some non-fall events are still detected as fall events [36]. To address this issue, Reference [37] recorded several types of daily activities (standing, walking, sitting and bending) and segmented them from several types of fall events (slipping, fainting and tripping). A hidden Markov model is used to analyze the frequency corresponding to the fall rate and is highly accurate. As vision-based and sound-based methods are obviously not preferred in the bathroom, Reference [38] proposed an interesting radar-based fall-detection method that consists of two stages: Prescreen and classification. The first stage identifies when a fall may occur and the second stage extracts the frequency signals from wavelet coefficients at many scales as features to classify. Their experiment showed that this method performs robustly in bathrooms. Due to the limitations of traditional machine-learning methods, Reference [39] proposed a radar-based method using deep learning and achieved high fall-detection accuracy. However, many radar-based methods are used in areas where the radar signal is not limited and the objects between the radar (such as pets and furniture) are not taken into consideration [40], resulting in these methods not being practical. Solving these problems may be interesting future work. The wireless-based fall detection method WiFall was proposed [41,42], which uses commercial Wi-Fi devices. The main idea with this method is to use the sharp power-profile-decline pattern to detect falls and its major advantage is that it detects falls with a commercial Wi-Fi device without the need for any other sensors or wearable devices. Although wireless-based methods are an exciting solution to fall detection, there are still problems with them. They are not robust against environmental changes and different types of or many people.

### 2.2. Wearable/Mobile-Device-Based Methods

Wearable-device-based fall-detection methods are roughly categorized into two types: Wearable-sensor based and wearable-camera based. The main advantage of these methods is that they work both indoors and outdoors. Wearable-sensor-based methods have been widely studied as they are low cost and non-invasive in terms of privacy. Traditional machine-learning-based fall-detection methods involving wearable sensors usually use a non-lapping [43] or overlapping [44,45] sliding window to segment the data then extract features to classify fall and non-fall events. However, these methods may lose useful information. Reference [46] proposed a machine-learning-based method that is triggered by a fall event. This method uses a buffer to restore samples as a pre-process then detects multiple peaks as the trigger time. By comparing the samples in the buffer, fall/non-fall events are classified. The computational cost of this method is just 80% that of a non-overlapping sliding-window-based method and 78% that of an overlapping sliding-window-based method. However, the accuracy is still not high enough for practical use. To address this issue, Reference [47] applied a Kalman filter to a non-liner filter to reduce the error rate of fall-event detection and Reference [48] collected actual data of elderly individuals and compared several methods to improve the fall-detection rate. Reference [49] developed a fog-computing-based deep-learning method and improved its performance by using a data-augmentation technique. Reference [50] developed an application for smart watches to collect the actual data of volunteers to train a fall-detection model and considered problems for practical use, for example, habits of elderly individuals and the last time of battery. As there are few publicly available data sets for sensor-based fall detection, the approach of Reference [51] creates an accelerometer and one gyroscope-based fall-detection data set, which is a well-constructed data set. Nineteen types of daily activities and 15 types of fall activities are included in this data set. Unlike other fall-detection data sets, the data of 14 actual elderly individuals over 60 years old are provided. The wearable-camera-based fall-detection methods proposed by References [52,53,54] involve fixing a camera to a waistband and detecting falls by analyzing the changes in the images taken with the camera. This is an interesting solution that combines the advantages of vision-based and wearable-sensor-based methods. However, it is weak in terms of privacy. As wearable-sensor-based methods can be used almost ubiquitously and the sensor is low cost, commercial wearable devices have been developed, such as Apple Watch 4 [55], Medical Guardian Fall Alert [56] and Philips Lifeline Auto Alert [57].

### 2.3. Pressure-Sensor-Based Methods

Although wearable/mobile-device based methods perform well, they may be weak at night. When an elderly individual goes to the toilet at night, she/he may forget to wear the smart sensor/watch or take his/her smart phone. In such a case, the elderly individual cannot be monitored [58] under weak light conditions at night [8]. Pressure-sensor-based methods provide a good solution to this problem since an elderly individual does not need to wear any device. The method proposed by Reference [59] tracks an individual by using NFI floor [60], then the features associated with that individual are extracted. We extracted the number of observations, longest dimension and sum of magnitudes as features and estimated the pose of the individual by using these features to detect falls. The method in Reference [58] involves using pressure mate sensors (PMSs) and passive infrared sensors (PISs) to detect falls. The actions of ten elderly individuals generated using a simulator were used to create a data set. When an individual has fallen, the PMS and PIS are turned OFF and the fall is detected. The method proposed in Reference [61] involves using fiber-optic sensors to detect falls. Pressure is detected with these sensors and a histogram is created to analyze human activity. If an individual is lying down longer than a threshold, a fall is detected. As pressure sensor-based methods cannot differentiate between falls and lying down for long periods, the method in Reference [62] uses the fusion between pressure sensors and accelerometers hidden under smart tiles to detect falls and improve fall-detection accuracy. Pressure-sensor-based methods are low cost, reliable and accurate but not easy to install and maintain. Solving these problems is for future work.

### 2.4. Vision-Based Methods

Vision-based fall-detection methods have recently been studied since users do not need to wear devices or charge batteries. When an elderly individual goes to the toilet at night, she/he may forget to put on the wearable device or charge the battery. Therefore, although camera-based methods are limited to a very restricted area, they are still useful for elderly individuals. Some studies categorized vision-based methods into two types—RGB-camera based and depth-camera based [8,29]. Depth-camera-based methods usually perform better since the depth camera is not affected by the changes in illumination; thus, works well in dark rooms, and does not invade privacy. Hence, depth-camera-based methods are gaining attention. However, many RGB-camera-based methods can be used to effectively process depth-based images. Therefore, we surveyed vision-based methods by categorizing them into two types based on the analysis method: skeleton and shape. Skeleton-tracking-based methods usually involve depth cameras to capture images, as it is easy to track the 3D joints of people. The methods proposed in References [23,24] obtain the 3D coordinates of joints and the ground-plane equation. Falls can be detected by analyzing their relationship. The method proposed in Reference [29] uses a support vector machine (SVM) to classify the 3D coordinates of joints during fall and non-fall events but only walking, sitting and falling are taken into consideration. The method proposed in Reference [25] extracts the changing rate of the torso angle as a feature by using 3D skeleton coordinates to classify falls from other fall-like activities. The method proposed in Reference [63] extracts similar features to predict fall risk. The accuracy of these methods is very high but the main drawback is that they heavily depend on the skeleton-detection results from Microsoft Kinet SDK or OpenNI and are not reliable enough [8]. Reference [28] proposed a head-tracking method but the camera is set at a very low position, which may be occluded by furniture. The methods proposed in References [25,29,63] face the same problem. Shape analyzing is also a good method for detecting falls. Bounding boxes and bounding-ellipse-based fall-detection methods were proposed more than a decade ago [11] and are still effective in detecting falls and have been improved [7,64]. The main drawback of these methods is that bounding boxes are heavily affected by the camera direction and shape analyses based on histogram analysis [15] have the same problem. Thus, multiple cameras are necessary to achieve high accuracy. To address this issue, fusion of data obtained from different cameras is an alternative and has been recently studied. The method in Reference [16] involves setting up four cameras and using voting algorithms to detect falls in different directions. To cover a larger range, multiple cameras have been used [12]. Three-dimensional shape analysis is a good solution to this issue [27] but incurs a high computational cost [65].

### 2.5. Discussion

Based on the results of our survey, we found the following problems to address and we design our algorithm and experiment.

Q1: Many studies fixed the camera at only one height. This limits the fall-detection method in terms of practicality since users may not set the camera at the same height. Does the camera height affect the fall-detection results? Is it possible to design a fall-detection method that is robust against camera height?

Since no data set is available to analyze how camera height affects fall-detection accuracy, this study creates a large data set consisting of 16 daily activities and 16 types of falls. Each activity is carried out by eight people in eight directions and taken with a depth camera at five different heights. About 350,000 images (frames) are included in this data set. This data set also includes human-segmentation results, which will be convenient for comparative analysis. Furthermore, these images are analyzed using a machine-learning method and deep-learning methods to study the effect of camera height on fall-detection accuracy.

Q2: Many studies were heavily based on the human/skeleton tracking results from Kinect SDK but they are not reliable enough. Is it possible to improve upon human tracking?

This study captures a large number of depth images and recorded the human tracking results of Kinect SDK (human segmentation). We then developed ETDA-Net to solve the problems found in these images and classify theme into fall and non-fall images.

Q3: Since users may set the camera at different heights, is it possible to have the system automatically detect camera height and select the most suitable model to initialize the fall-detection method?

This study designs an algorithm to automatically detect the height of the camera and select the most suitable model to detect falls.

## 3. Fall-Detection Data Set

A data set is important for vision-based fall-detection methods. However many have limitations as mentioned above:

The data set created in Reference [31] includes seven groups of images. The main contribution of this data set is that images taken in different rooms, for example, offices and coffee rooms, are provided. However, only a single camera was used with this data set and the camera height were not mentioned in this study. The data set created in Reference [32] includes depth images but there are only 21,499 images. That created in Reference [33] is a well-constructed data set and 12 people participated in their experiment. However, the camera was set at only one height, which was too low to avoid occlusion problems. The data set created in Reference [34] is also well-constructed and includes 30 fall videos and 40 videos of daily activities. Synchronization and accelerometer data are also recorded. However, the camera was set at a very low position, which may be occluded by furniture (although another camera was set on the ceiling, it only recorded fall videos) in their experiment.

**Figure 1:**
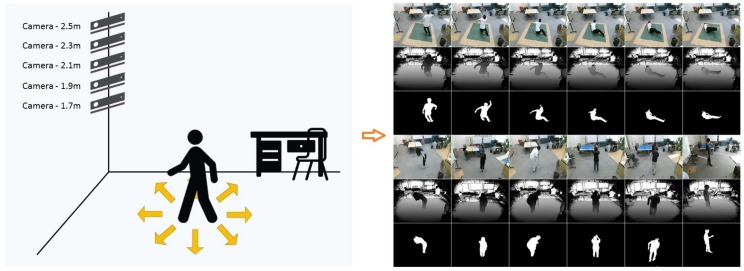
Diagram of our data set.

The images in our data set to study the effect of camera height on fall-detection accuracy are captured using a Kinect V2 depth sensor linked to a notebook PC. The CPU of this notebook PC is Intel core i5 5200 u and the memory is 4 GB. The frame rate is 20 fps. As mentioned above, this data set provides depth images and consists of 16 daily activities (walking, running, kicking, bowing, bending, walking with a stoop, clipping, rising hands, waving hands, looking at a watch, using a smartphone, throwing, drinking water, collecting, walking with a stick, sitting on the chair/floor) and 16 types of falls (facing ceiling, floor, left, right, with/without curled up legs, with left/right hand moving), as shown in Table 1. Facing floor means the final state of the fall is the person ending up facing the floor (facing ceiling, left, right have the similar respective meanings). Each activity is carried out by eight people in eight directions and taken using a camera at five different heights (1.7, 1.9, 2.1, 2.3 and 2.5 m). Each activity is repeated 2–3 times at each direction. A total of 800 streams (640 non-fall streams and 160 fall streams) with about 350,000 depth images (frames) are included in this data set. This data set also includes human segmentation results, which will be convenient for comparative analysis, as shown in Figure 1. This data set is publicly available [66] and the information of 8 participants is shown in Table 2.

## 4. Fall Detection System

### 4.1. Overview

**Figure 2:**
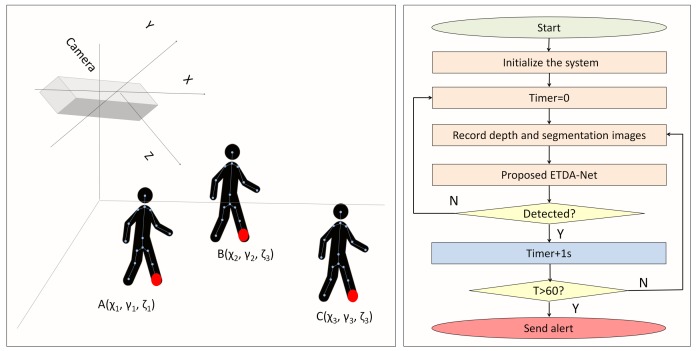
Procedure of Enhanced Tracking and Denoising Alex-Net (ETDA-Net).

The proposed method consists of three steps—system initialization, human segmentation and fall detection. When a fall is detected, a timer starts. If the classification result is “fall” and this situation lasts more than one minute, an alert is sent to the hospital, health center or family members. Total silence of the participant is not required, as elderly individuals may move on the floor due to pain. If there are two or more individuals in a room, this system does not make a classification (since Kinect can track and segment six different individuals, this situation can be recognized). This section gives a detailed introduction of these steps, which are also illustrated in Figure 2.

### 4.2. System Initialization

Nowadays, users may set the cameras at different heights. Therefore, it is important to have the fall-detection method calculate the camera height and initialize it to improve fall-detection accuracy. The basic idea of this method is regarding the camera as a point and the floor as a plane. Camera height is determined by calculating the distance from the point to the plane. The first step is marking three points on the floor and obtaining their 3D coordinates. This is possible by capturing the 3D coordinates of the ankle joint three times because this joint is very close to the floor, as shown in Figure 2. Points $A(\chi_{1},\gamma_{1},\zeta_{1})$, $B(\chi_{2},\gamma_{2},\zeta_{2})$, $C(\chi_{3},\gamma_{3},\zeta_{3})$ give the 3D position of the ankle joint in different places. Therefore, a normal vector $\vec{NV}$ is given by Formula (1) and this vector is perpendicular to the plane (2). The coefficients of i,j and *k* in this vector, are the coefficients of the plane equation of the floor. The coefficients of i,j and *k* are defined as *a*,*b* and *c*,as given in Formulas (3)–(5). The plane equation of the floor is given by Formula (2) and parameters χ, γ and ζ are on a three coordinate axis. Point $N(\chi_{0},\gamma_{0},\zeta_{0})$ is the coordinate of camera N and the distance from the camera to the ground plane *D* is given by Formula (7). As the camera is at the origin of the coordinates, system sets χ, γ and ζ to zero to calculate the distance from the camera to the floor.

(1) $$\vec{NV}=\left[\begin{array}{ccc} \vec{i} & \vec{j} & \vec{k}\\ -\chi_{1}+\chi_{2} & -\gamma_{1}+\gamma_{2} & -\zeta_{1}+\zeta_{2}\\ -\chi_{1}+\chi_{3} & -\gamma_{1}+\gamma_{3} & -\zeta_{1}+\zeta_{3} \end{array}\right]$$

(2) $$\left[\begin{array}{ccc} \chi -\chi_{1} & \gamma -\gamma_{1} & \zeta -\zeta_{1}\\ \chi_{2}-\chi_{1} & \gamma_{2}-\gamma_{1} & \zeta_{2}-\zeta_{1}\\ \chi_{3}-\chi_{1} & \gamma_{3}-\gamma_{1} & \zeta_{3}-\zeta_{1} \end{array}\right]=0$$

(3) $$a=\left[\begin{array}{cc} \gamma_{2}-\gamma_{1} & \zeta_{2}-\zeta_{1}\\ \gamma_{3}-\gamma_{1} & \zeta_{3}-\zeta_{1} \end{array}\right]$$

(4) $$b=\left[\begin{array}{cc} \chi_{3}-\chi_{1} & \zeta_{3}-\zeta_{1}\\ \chi_{2}-\chi_{1} & \zeta_{2}-\zeta_{1} \end{array}\right]$$

(5) $$c=\left[\begin{array}{cc} \chi_{2}-\chi_{1} & \gamma_{2}-\gamma_{1}\\ \chi_{3}-\chi_{1} & \gamma_{3}-\gamma_{1} \end{array}\right]$$

(6) $$d=\chi_{1}\left[\begin{array}{cc} \gamma_{3}-\gamma_{1} & \zeta_{3}-\zeta_{1}\\ \gamma_{2}-\gamma_{1} & \zeta_{2}-\zeta_{1} \end{array}\right]+\gamma_{1}\left[\begin{array}{cc} \chi_{2}-\chi_{1} & \zeta_{2}-\zeta_{1}\\ \chi_{3}-\chi_{1} & \zeta_{3}-\zeta_{1} \end{array}\right]+\zeta_{1}\left[\begin{array}{cc} \chi_{3}-\chi_{1} & \gamma_{3}-\gamma_{1}\\ \chi_{2}-\chi_{1} & \gamma_{2}-\gamma_{1} \end{array}\right]$$

(7) $$D=\frac{\left|a\chi_{0}+b\gamma_{0}+c\zeta_{0}+d\right|}{\sqrt{a^{2}+b^{2}+c^{2}}}$$

### 4.3. Human Segmentation and Fall Detection

Generally speaking, the first step in fall-detection methods is human segmentation. Human segmentation based on Kinect SDK [20] is considered good for segmenting the human body and many studies heavily depended on the segmentation results (including skeleton tracking) of Kinect SDK. A depth camera captures depth images sequentially and the features of these images are calculated using Formula (8).

(8) $$G_{\delta }\left(P,i\right)=d_{P}\left(i+\frac{m}{d_{P}\left(i\right)}\right)-d_{P}\left(i+\frac{n}{d_{P}\left(i\right)}\right)$$

Parameter $G_{\delta }\left(P,i\right)$ is the depth comparison features at pixel *i* in depth image *P* [67]. Parameter *m* and *n* are offsets and vector δ, which is calculated by (m,n), describes 2D pixel offsets in the depth image *P* and $d_{P}\left(i\right)$ is the depth value at pixel *i* in *P*. During the training process, offsets *m* and *n* are sampled at random within a box of fixed size. After feature extraction, randomized decision forests are used to cluster these features [20]. Parameter *l* is the human-body-part label and by using the final result of clustering $R_{b}\left(l|P,i\right)$ [68], the body parts of individuals are detected; *b* is the tree label. In other words, the binary images of individuals are created by setting the pixel values of the body parts to 255. Refer to Reference [68] for more details.

**Figure 3:**
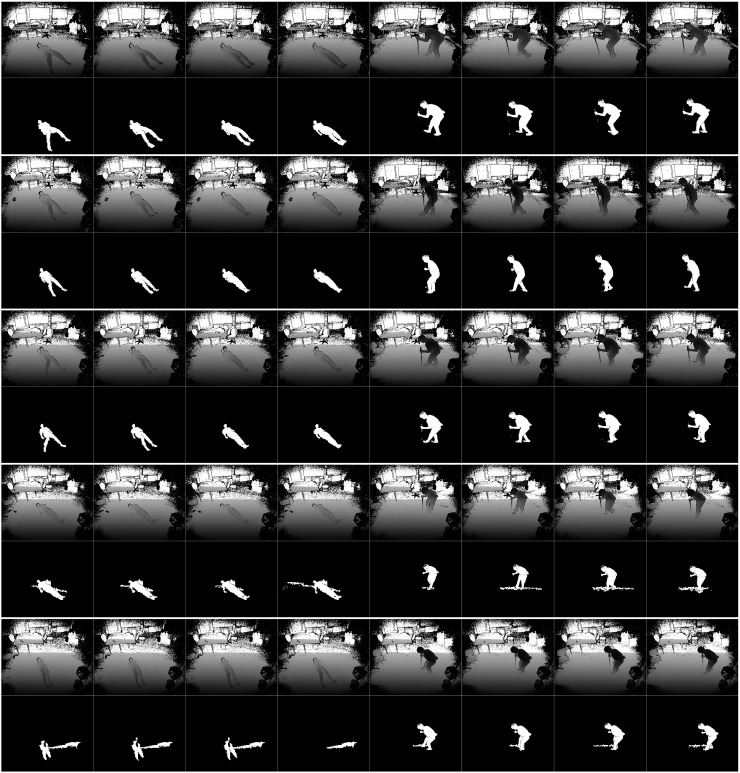
Human segmentation using Kinect SDK. Five lines are human segmentation results from Kinect set at 1.7, 1.9, 2.1, 2.3 and 2.5 m.

However, this method is not reliable enough for a fall detection. As shown in Figure 3, there are five rows in this figure. Each rows contains two groups of images. At each row, the four left images are fall events and the four right images are non-fall events. The images above are depth images and the images below are human-segmentation images. According to the experimental results, if the depth camera is set at a low position (1.7, 1.9 or 2.1 m), it segments the person well—the background is removed completely and only the individual is left in the image. However, when the camera is set at 2.3 or 2.5 m, two serious problems occur—wide range noise and tracking loss. Setting the camera at a low position is obviously not preferred for practical use as it may be often occluded by furniture.

**Figure 4:**
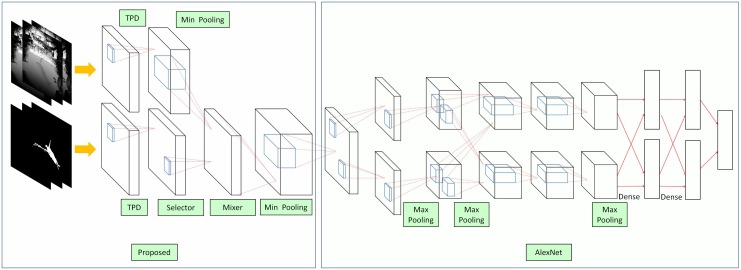
ETDA-Net.

To address this issue, ETDA-Net segments the human body and detects falls, as shown in Figure 4. The ETDA-Net contains two parts—pre-processing (left) and a convolutional neural network (CNN) (right). The pre-processing part reduces the noise in the images and improves tracking performance and the CNN part is the same as with AlexNet [69] and the hyper-parameters are optimized automatically by NVIDIA DIGITS 6 [70]. This net uses the depth and human-segmentation images as input, improving segmentation performance. The main idea with ETDA-Net is to compare the partial derivative of depth images to that of segmentation images. If the pixels of segmentation images have a sharp change but the corresponding pixels in the depth images do not, these pixels are considered as tracking loss or noise. Therefore, this study proposes two time partial derivative (TDP) layers to obtain the partial derivative of depth images and segmentation images. This study then uses a min pooling layer to reduce the noise in the derivative depth images. This study uses a selector layer to confirm if tracking loss occurs and a mix layer to add the tracking results to the segmentation images if tracking loss is detected. Finally, a min pooling layer is employed to reduce the noised in the mixed images and these images are sent to Alex Net for classification.

A TDP layer calculates the partial derivative depth images and segmentation images, which are given by Formula (9), where ${\hat{O}}_{\left(m,n\right)}$ is the output value of the pixel at point (m,n), $G(P_{\left(m,n,t\right)})$ is the value of the pixel at point (m,n) at time *t* and $\frac{\partial G(P_{\left(m,n,t\right)}))}{\partial t}$ denotes how the pixel changes along with coordinates and time. As two depth images and two segmentation images are input into the net at the same time, each pixel is four dimensions and the input size is 512 × 424.

(9) $$\left[\begin{array}{cccc} {\hat{O}}_{\left(0,0\right)} & {\hat{O}}_{\left(0,1\right)} & \cdots & {\hat{O}}_{\left(0,n\right)}\\ {\hat{O}}_{\left(1,0\right)} & {\hat{O}}_{\left(1,1\right)} & \cdots & {\hat{O}}_{\left(1,n\right)}\\ \cdots & \cdots & \cdots & \cdots \\ {\hat{O}}_{\left(m,0\right)} & {\hat{O}}_{\left(m,1\right)} & \cdots & {\hat{O}}_{\left(m,n\right)} \end{array}\right]=\left[\begin{array}{cccc} \frac{\partial G(P_{\left(0,0,t\right)}))}{\partial t} & \frac{\partial G(P_{\left(0,1,t\right)}))}{\partial t} & \cdots & \frac{\partial G(P_{\left(0,n,t\right)}))}{\partial t}\\ \frac{\partial G(P_{\left(1,0,t\right)}))}{\partial t} & \frac{\partial G(P_{\left(1,1,t\right)}))}{\partial t} & \cdots & \frac{\partial G(P_{\left(1,n,t\right)}))}{\partial t}\\ \cdots & \cdots & \cdots & \cdots \\ \frac{\partial G(P_{\left(m,0,t\right)}))}{\partial t} & \frac{\partial G(P_{\left(m,1,t\right)}))}{\partial t} & \cdots & \frac{\partial G(P_{\left(m,n,t\right)}))}{\partial t} \end{array}\right]$$

Since random noise occurs in the depth images at the range of the object, the min pooling layer is designed to reduce such noise. This layer is described by Formula (10), where $\hat{P}\left(x,y\right)$ is the value of the output pixel at point (x,y), τ(x), τ(y) is filter range and $\hat{P}\left(\bar{x},\bar{y}\right)$ is a filter window surrounding pixel(x,y). Parameters τ(x), τ(y) are optimized using the control-variates method and are set to 5 × 5 in our study.

(10) $$\hat{P}\left(x,y\right)=min_{\bar{x}\in \tau \left(x\right),\bar{y}\in \tau \left(y\right)}\hat{P}\left(\bar{x},\bar{y}\right)$$

The selector layer determines how to mix the lost pixels (remove noise or correct tracking loss) in the mixer layer given by Formula (11), where Ls is the number of lost pixels and Le is the number of pixels left in the image. Parameter *t* is a control threshold which is optimized by Ls and Le of training images. When *M* is set to 0, the mix layer only remains noise redaction image and when *M* is set to 1, the mix layer mixes the partial derivative depth image to the segment image in order to correct tracking loss, as shown in Formula(12), where $G(P_{\left(m,n,t\right)})$ is the value of the pixel at point (m,n) at time *t* and $\frac{\partial G(P_{\left(m,n,t\right)}))}{\partial t}$ denotes how the pixel changes along with coordinates and time and $I_{M}$ is the output of the mixed layer. Since this process may cause noise in the image, another min pooling layer is used to reduce such noise.

(11) $$M=\frac{1}{1+e^{-t(Ls/Le)}}$$

(12) $$I_{M}=\left[\begin{array}{cccc} {\hat{S}}_{\left(0,0\right)} & {\hat{S}}_{\left(0,1\right)} & \cdots & {\hat{S}}_{\left(0,n\right)}\\ {\hat{S}}_{\left(1,0\right)} & {\hat{S}}_{\left(1,1\right)} & \cdots & {\hat{S}}_{\left(1,n\right)}\\ \cdots & \cdots & \cdots & \cdots \\ {\hat{S}}_{\left(m,0\right)} & {\hat{S}}_{\left(m,1\right)} & \cdots & {\hat{S}}_{\left(m,n\right)} \end{array}\right]+M*\left[\begin{array}{cccc} \frac{\partial G(P_{\left(0,0,t\right)}))}{\partial t} & \frac{\partial G(P_{\left(0,1,t\right)}))}{\partial t} & \cdots & \frac{\partial G(P_{\left(0,n,t\right)}))}{\partial t}\\ \frac{\partial G(P_{\left(1,0,t\right)}))}{\partial t} & \frac{\partial G(P_{\left(1,1,t\right)}))}{\partial t} & \cdots & \frac{\partial G(P_{\left(1,n,t\right)}))}{\partial t}\\ \cdots & \cdots & \cdots & \cdots \\ \frac{\partial G(P_{\left(m,0,t\right)}))}{\partial t} & \frac{\partial G(P_{\left(m,1,t\right)}))}{\partial t} & \cdots & \frac{\partial G(P_{\left(m,n,t\right)}))}{\partial t} \end{array}\right]$$

After tracking correction and noise reduction, the images are classified as the left layer of this net, which is the same with traditional deep-learning-based fall-detection methods [71].

## 5. Experimental Results

### 5.1. Experimental Results of System Initialization

The main process of system initialization is automatically calculating camera height. The experimental results are listed in Table 3. The proposed method accurately detected camera height. The height is a little lower than the actual height because this method uses the coordinates of ankle joints instead of those of the actual floor. This work measure the ankle height of different subjects and finds out the average height is 9.2 cm. Therefore, this work gives a correction parameter to measure the height more rigorously.

### 5.2. Experimental Results from Human Segmentation

**Figure 5:**
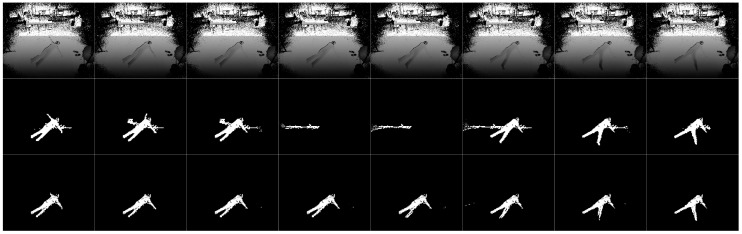
Experimental results of human segmentation. First row is depth images, second row is human segmentation using Kinect SDK and the third row is human segmentation using ETDA-Net.

The results of human segmentation are shown in Figure 5. The first row shows the depth images, the second row shows the human segmentation results of Kinect SDK and the third row shows the human segmentation results using ETDA-Net. ETDA-Net produced better results in both human tracking and denoising.

### 5.3. Parameters and Performance-Evaluation Metrics

The eight participants in the experiment are divided into four groups. Participants A and B are in group 1, C and D in group 2, E and F in group 3 and G and H in group 4. During each test, three groups are for training and the remaining group is for testing to make the test data different with the training data. Then The data are trained using a desktop PC. The operation system is Ubuntu 16.04, the CUP is Intel(R) Xeon(R) CPU E5-1650 v4 3.60GHz and the GPUs are triple NVIDIA GTX 1080Ti with 33-GB memory. The framework is Torch and the hyper-parameters of the CNN are optimized automatically by NVIDIA DIGITS 6 [70]. The window size of 64 × 128, block size of 16 × 16, block stride of 8 × 8 and a cell size of 8 × 8 (only these sizes are supported at OpenCV) [72] are set as the parameters of histogram of oriented gradient (HOG) features. A totally of 800 video streams with 240,000 images (frames) are used in the experiment (not all the frames are used since the fall streams contained non-fall frames), while 180,000 images (frames) are used for training and 60,000 are used for testing. At each height, 6000 fall states (frames) and 6000 non-fall states (frames) are included. The shape of a participant does not change with distance but the size does. Therefore, the images of the participants are normalized to 256 × 256 (for AlexNet, GoogLeNet and ETD-AlexNet) or 28 × 28 (for LeNet) before input to the neural network.

We used the following performance-evaluation metrics proposed in Reference [73] to analyze the experimental results.

**True positive (TP)** is the number of fall events accurately detected.

**True negative (TN)** is the number of non-fall events accurately detected.

**False positive (FP)** is the number of non-fall events detected as fall events.

**False negative (FN)** is the number of fall events detected as non-fall events.

In the study in Reference [73], sensitivity (Se), specificity (Sp), accuracy (Ac) and error (Er) are given by the following formulas to obtain the performance-evaluation metrics:

(13) Se=TP/(TP+FN)

(14) Sp=TN/(TN+FP)

(15) Ac=(TP+TN)/(TP+TN+FP+FN)

(16) Er=(FP+FN)/(TP+TN+FP+FN)

### 5.4. Effect of Camera Height and Comparison

Table 4, Table 5 and Table 6 compare the experimental results of sensitivity, specificity and accuracy, respectively. The Training data column means the camera height at which training images are taken and the Test data column means the camera height at which test images are taken. The performance of ETDA-Net is compared with the machine-learning method HOG+SVM [74] and deep-learning methods LeNet [75], AlexNet [71] and GoogLeNet [76]. The effect of camera height on fall-detection accuracy is clearly illustrated in these tables. The fall-detection accuracy is high if the training images and test images are taken with the camera at the same height. However, the more this differs, the lower the accuracy. Therefore, camera height is an important parameter for fall-detection methods, which has been ignored in many studies.

Several studies set the camera at a low position. If the user sets the camera at a higher position, according to the experimental results, fall detection accuracy will drastically reduce, especially when the camera is set at a height of over 2.3 m.

According to these tables, the accuracy of HOG+SVM decreased by 38.4%, 17.5%, 20.2%, 25.9% and 31.9% at 1.7, 1.9, 2.1, 2.3 and 2.5 m, respectively, that of LeNet decreased by 50.5%, 12.1%, 35.7%, 31.1% and 50.4%, that of AlexNet decreased by 19.3%, 5.4%, 28.9%, 3.2% and 24.9%, that of GoogLeNet decreased by 10.7%, 4.2%, 21.5%, 2.3% and 29.2% and that of ETDA-Net decreased by 15.7%, 2.9%, 13.7%, 0.3% and 17.3% (the farthest distance is treated as a reference). ETDA-Net exhibited the most robust performance at 1.9, 2.1, 2.3 and 2.5 m. It only takes about 1.3 h to train ETDA-Net, while the training time of GoogLeNet is more than 8 h (trained by all the available data for each group). This lowered the computational cost of ETDA-Net compared to GoogLeNet, whose performance is similar to that of ETDA-Net. When the model is trained by all the available data, the robust performance is the best but is still less than 100%. Therefore, if the camera height is accurately calculated, ETDA-Net trained by the same height provides good fall-detection results.

### 5.5. Discussion and Future Work

Training on all or some images: As shown in Table 4, Table 5 and Table 6, when the model is trained on all the available data, ETDA-Net exhibits the best robust performance. When the model is trained on only one height, the accuracy is higher (only when the camera height is accurately calculated) but performance becomes less robust. Considering the effect of furniture/pets, the camera height may not be accurately calculated; therefore, training the model with images at different heights may be a robust and credible solution at the current state. Developing an algorithm to calculate the camera height more efficiently and reliably will be for future work. This study confirms that training the model on images taken at different heights or calculating the camera height and selecting the model is necessary.

Sensitivity or specificity: Although ETDA-Net provides good results, it is still not unquestionably better than GoogLeNet: ETDA-Net has higher specificity, so it raises less false alarms; However, sometimes it has lower sensitivity (e.g., for the training data acquired at 1.7 m), so it ignores more actual falls. In other words, the sensitivity of a fall-detection method is more important because each non-detected fall may lead to death, while a false alarm may only lead to an unnecessary visit of a health-care personnel. Therefore, improving the sensitivity of ETDA-Net will be interesting future work.

Camera orientation: Since the monitoring angle (depth) of Kinect is only 70 degrees (horizontal) and 60 degrees (vertical), a large room cannot be monitored [30], so the camera orientation is difficult to analysis; Therefore, it is set manually in our study. If the sensor is set at a high place (no less than 2.0 m) and the angle between the wall and main optical axis is more than 23.75 degrees, a larger range can be monitored [30]. On the other hand, when the camera is placed parallel to the subject or up to 35 degrees higher, the accuracy is higher with the method in Reference [26]. However, as the monitoring angle of Kinect is not wide enough, the analysis of camera orientation makes little sense in our study. Therefore, the development of a wide-angle depth camera and analysis of the orientation of this camera will be for future work.

## 6. Conclusions

This study focuses on the effect of camera height on fall-detection methods, which has not been extensively studied. Since there is no open data set for analyzing camera height, this study creates a large data set that includes about 350,000 depth images. For this data set, 16 types of daily activities and 16 types of falls are carried out by eight people in eight directions and captured with a depth camera at five different heights. This study also introduces an algorithm for automatically calculating camera height, making it possible for the system to adapt to the environment. This study also proposes a two-channel input neural-network-based called ETDA-Net to improve the human segmentation performance of Kinect SDK and classify fall and non-fall events. The experimental results indicate that ETDA-Net outperforms traditional machine-learning and deep-learning methods and is robust against different heights. For future work, we plan to study the impact of the different behaviors of individuals on fall-detection accuracy and develop a system for automatically analyzing such behavior and selecting the most suitable model to use to detect falls. We will also develop an algorithm to calculate the camera height more efficiently and reliably and improve sensitivity. 

## Figures and Tables

**Table 1:** Evaluation scenarios in data set.

Fall Events	Non-fall Events
Facing left, with curled up legs	walking
Facing left, without curled up legs	running
Facing right, with curled up legs	kicking
Facing right, without curled up legs	bowing
Facing floor, with curled up legs	bending
Facing floor, without curled up legs	walking with stoop
Facing ceiling, with curled up legs	clipping
Facing ceiling, without curled up legs	raising hand
Facing left, with left hand moving	waving hand
Facing left, with right hand moving	looking at watch
Facing right, with left hand moving	using smartphone
Facing right, with right hand moving	throwing
Facing floor, with left hand moving	drinking water
Facing floor, with right hand moving	collecting
Facing ceiling, with left hand moving	walking with stick
Facing ceiling, with right hand moving	sitting on chair/floor

**Table 2:** Information of 8 participants.

Participant ID	Gender	Age	Weight [kg]	Height [cm]
A	Male	24	89	172
B	Female	24	52	161
C	Male	22	93	173
D	Male	29	72	178
E	Female	23	48	158
F	Male	25	60	167
G	Male	24	63	171
H	Female	24	65	160

**Table 3:** Experimental results from camera-height detection.

**Camera Height [m]**	1.70	1.90	2.10	2.30	2.50
**Detected Height [m]**	1.62	1.83	2.03	2.23	2.43
**After Correction [m]**	1.71	1.92	2.12	2.32	2.52

**Table 4:** Experimental results from sensitivity comparison.

Training Data	Test Data	HOG+SVM	LeNet	AlexNet	GoogLeNet	ETDA-Net
1.7 m	1.7 m	**100.0%**	**100.0%**	**100.0%**	**100.0%**	**100.0%**
	1.9 m	88.2%	80.6%	87.0%	**93.8%**	89.4%
	2.1 m	92.0%	99.0%	99.0%	**100.0%**	99.6%
	2.3 m	43.6%	23.0%	66.0%	**93.2%**	61.2%
	2.5 m	47.2%	15.8%	69.0%	**93.8%**	76.6%
1.9 m	1.7 m	97.0%	95.0%	99.6%	**100.0%**	**100.0%**
	1.9 m	99.8%	**100.0%**	**100.0%**	**100.0%**	**100.0%**
	2.1 m	**100.0%**	**100.0%**	**100.0%**	**100.0%**	**100.0%**
	2.3 m	74.4%	85.8%	91.4%	**97.6%**	96.4%
	2.5 m	81.2%	81.2%	92.0%	**96.4%**	95.6%
2.1 m	1.7 m	88.0%	94.8%	77.4%	**97.6%**	92.6%
	1.9m	**97.2%**	91.4%	85.6%	92.8%	90.8%
	2.1 m	99.8%	**100.0%**	**100.0%**	**100.0%**	**100.0%**
	2.3 m	**80.0%**	70.4%	68.8%	82.0%	72.8%
	2.5 m	**83.2%**	30.0%	42.8%	57.6%	72.8%
2.3 m	1.7 m	47.4%	37.2%	93.6%	95.2%	**99.2%**
	1.9 m	83.2%	90.8%	99.4%	98.8%	**100.0%**
	2.1 m	89.2%	100.0%	100.0%	98.6%	**100.0%**
	2.3m	99.8%	99.8%	**100.0%**	100.0%	**100.0%**
	2.5 m	97.0%	69.8%	**100.0%**	96.6%	99.8%
2.5 m	1.7 m	35.8%	0.0%	51.6%	40.6%	**65.2%**
	1.9 m	80.4%	46.4%	**90.2%**	55.6%	88.4%
	2.1 m	91.4%	54.2%	67.8%	50.4%	**96.4%**
	2.3 m	98.0%	98.6%	97.4%	79.6%	**100.0%**
	2.5 m	100.0%	99.8%	**98.8%**	**98.2%**	**100.0%**
All	1.7 m	-	93.7%	98.4%	98.9%	99.2%
	1.9 m	-	99.8%	99.9%	99.8%	**100.0%**
	2.1 m	-	96.2%	**99.9%**	99.7%	99.4%
	2.3 m	-	99.7%	99.8%	**100.0%**	99.8%
	2.5 m	-	99.6%	99.5%	**99.7%**	99.6%

**Table 5:** Experimental results from specificity comparison.

Training Data	Test Data	HOG+SVM	LeNet	AlexNet	GoogLeNet	ETDA-Net
1.7 m	1.7 m	**100.0%**	**100.0%**	**100.0%**	**100.0%**	**100.0%**
	1.9 m	99.0%	99.4%	**100.0%**	**100.0%**	**100.0%**
	2.1 m	99.6%	99.4%	99.8%	99.4%	**100.0%**
	2.3 m	95.4%	94.8%	98.2%	97.2%	**99.0%**
	2.5 m	76.0%	83.2%	**92.4%**	84.8%	92.0%
1.9 m	1.7 m	99.6%	**100.0%**	**100.0%**	99.8%	**100.0%**
	1.9 m	**100.0%**	99.8%	**100.0%**	**100.0%**	**100.0%**
	2.1 m	99.8%	98.6%	**100.0%**	**100.0%**	**100.0%**
	2.3m	96.8%	97.6%	99.2%	98.8%	**99.6%**
	2.5 m	83.6%	94.4%	97.2%	95.2%	**98.6%**
2.1 m	1.7 m	99.4%	**100.0%**	**100.0%**	**100.0%**	**100.0%**
	1.9 m	97.2%	**100.0%**	**100.0%**	**100.0%**	**100.0%**
	2.1 m	**100.0%**	**100.0%**	**100.0%**	**100.0%**	**100.0%**
	2.3 m	94.4%	99.4%	99.8%	**100.0%**	**100.0%**
	2.5 m	76.2%	98.6%	99.4%	99.4%	**99.8%**
2.3 m	1.7 m	**100.0%**	**100.0%**	**100.0%**	**100.0%**	**100.0%**
	1.9 m	96.4%	97.0%	97.4%	99.0%	**99.4%**
	2.1 m	96.4%	98.0%	99.0%	99.8%	**100.0%**
	2.3 m	99.4%	99.6%	**100.0%**	99.8%	99.8%
	2.5 m	99.4%	**99.6%**	99.4%	99.6%	99.4%
2.5 m	1.7 m	**100.0%**	98.6%	97.2%	99.0%	**100.0%**
	1.9 m	94.0%	89.2%	82.0%	**99.2%**	97.0%
	2.1 m	89.2%	88.8%	82.0%	98.4%	**99.4%**
	2.3 m	94.4%	94.0%	96.2%	99.8%	**100.0%**
	2.5 m	99.6%	99.6%	**99.8%**	**99.8%**	**99.8%**
All	1.7 m	-	98.9%	99.8%	99.7%	**100.0%**
	1.9 m	-	99.7%	99.8%	99.8%	**100.0%**
	2.1 m	-	98.9%	99.1%	99.7%	**100.0%**
	2.3 m	-	97.3%	98.3%	98.4%	**99.1%**
	2.5 m	-	94.5%	94.5%	**96.9%**	96.3%

**Table 6:** Experimental results from accuracy comparison.

Training Data	Test Data	HOG+SVM	LeNet	AlexNet	GoogLeNet	ETDA-Net
1.7 m	1.7 m	**100.0%**	**100.0%**	**100.0%**	**100.0%**	**100.0%**
	1.9 m	93.6%	90.0%	93.5%	**96.9%**	94.7%
	2.1 m	95.8%	99.2%	99.4%	99.7%	**99.8%**
	2.3 m	69.5%	58.9%	82.1%	**95.2%**	80.1%
	2.5 m	61.6%	49.5%	80.7%	**89.3%**	84.3%
1.9 m	1.7 m	98.3%	97.5%	99.8%	99.9%	**100.0%**
	1.9 m	99.9%	99.9%	**100.0%**	**100.0%**	**100.0%**
	2.1 m	99.9%	99.3%	**100.0%**	**100.0%**	**100.0%**
	2.3 m	85.6%	91.7%	95.3%	**98.2%**	98.0%
	2.5 m	82.4%	87.8%	94.6%	95.8%	**97.1%**
2.1 m	1.7 m	93.7%	97.4%	88.7%	**98.8%**	96.3%
	1.9 m	**97.2%**	95.7%	92.8%	96.4%	95.4%
	2.1 m	99.9%	**100.0%**	**100.0%**	**100.0%**	**100.0%**
	2.3 m	87.2%	84.9%	84.3%	**91.0%**	86.4%
	2.5 m	79.7%	64.3%	71.1%	78.5%	**86.3%**
2.3 m	1.7 m	73.7%	68.6%	96.8%	97.6%	**99.6%**
	1.9 m	89.8%	93.9%	98.4%	98.9%	**99.7%**
	2.1 m	92.8%	99.0%	99.5%	99.2%	**100.0%**
	2.3 m	99.6%	99.7%	**100.0%**	99.9%	99.9%
	2.5 m	98.2%	84.7%	**99.7%**	98.1%	99.6%
2.5 m	1.7 m	67.9%	49.3%	74.4%	69.8%	**82.6%**
	1.9 m	87.2%	67.8%	86.1%	77.4%	**92.7%**
	2.1 m	90.3%	71.5%	74.9%	74.4%	**97.9%**
	2.3 m	96.2%	96.3%	96.8%	89.7%	**100.0%**
	2.5 m	99.8%	99.7%	99.3%	99.0%	**99.9%**
All	1.7 m	-	96.3%	99.1%	99.3%	**99.6%**
	1.9 m	-	99.7%	99.8%	99.8%	**100.0%**
	2.1 m	-	97.6%	99.5%	99.7%	**99.7%**
	2.3 m	-	98.5%	99.0%	99.2%	**99.5%**
	2.5 m	-	95.5%	97.0%	98.3%	**97.9%**

## Abbreviations

The following abbreviations are used in this manuscript:

ETDA-Net	Enhanced Tracking and Denoising Alex-Net
HOG	Histogram of Oriented Gradient
SVM	Support Vector Machine
